# Sexual dysfunction among men with diabetes; a cross-sectional study at a specialised diabetes clinic in Sri Lanka

**DOI:** 10.1186/s12902-022-01108-1

**Published:** 2022-08-17

**Authors:** Nipun Lakshitha de Silva, Tharaka Athukorala, Jayathra Liyana Gamage, Gaya Katulanda, Prasad de Silva, Manilka Sumanatilleke, Noel Somasundaram

**Affiliations:** 1 Department of Clinical Sciences, Faculty of Medicine, General Sir John Kotelawala Defence University, Colombo, Sri Lanka; 2 Diabetes and Endocrine Unit, National Hospital of Sri Lanka, Colombo, Sri Lanka; 3 Department of Radiology, National Hospital of Sri Lanka, Colombo, Sri Lanka; 4 Department of Chemical Pathology, National Hospital of Sri Lanka, Colombo, Sri Lanka

**Keywords:** Sexual dysfunction, Erectile dysfunction, Diabetes mellitus

## Abstract

**Background:**

Male sexual dysfunction in diabetes is often an unrevealed clinical issue. Though many publications report the prevalence, there is limited data on its associations, impact, and health-seeking behaviour. The objectives were to assess the prevalence of male sexual dysfunction, its associations, impact and treatment-seeking among men with diabetes in a selected tertiary care Diabetes Clinic.

**Methods:**

A cross-sectional study was conducted at the Diabetes Clinic, National Hospital of Sri Lanka, from January to September 2020. Men with diabetes aged 18 to 70 years undergoing annual assessment were recruited consecutively. Socio-demographic and clinical information were collected using an interviewer-administered questionnaire. Erectile dysfunction (ED), premature ejaculation, mental health and quality of life were assessed using validated self-administered questionnaires. Cardiovascular autonomic reflex tests and total testosterone levels were performed. Penile colour Doppler ultrasonography was performed on consenting participants with erectile dysfunction. Associations were assessed using the chi-square test or Fisher’s exact for dichotomous variables and independent sample t-test for continuous variables.

**Results:**

Two hundred and twelve participants were recruited with a mean age of 54.1 (SD = 10.1) years. Erectile dysfunction was present in 168 (79.2%), (mild: 45, mild-moderate: 56, moderate: 26, severe: 41). Premature ejaculation was present in 26 (18.7%). Libido was low among 16%. Sexual dysfunction was not revealed to a health provider by 85.6% despite 60.5% experiencing psychological and/or relationship effects. Out of 18 who sought treatment, only 4 achieved a good response. Mean age (55.4 ± 9.5 vs 48.7 ± 10.6 years, *p* < 0.001) and duration of diabetes (10.9 ± 7.6 vs 5.8 ± 4.6 years, *p* < 0.001) were higher while eGFR was lower (73.9 ± 27.7 vs 100.51 ± 28.08 years, *p* < 0.008) among those with ED compared to those without. Diabetic retinopathy (4% vs 42%, *p* < 0.001), peripheral neuropathy (17.9% vs 38.4%, *p* = 0.041) and lower limb arterial disease (0% vs 12.2%, *p* = 0.04) were associated with ED. Arterial insufficiency was seen among 50% of the participants who underwent penile colour Doppler ultrasonography.

**Conclusions:**

Male sexual dysfunction is a pervasive yet underappreciated problem in diabetes care despite its effect on the individual. Patient and disease characteristics would guide the identification of high-risk individuals for targeted screening in clinical practice.

**Supplementary Information:**

The online version contains supplementary material available at 10.1186/s12902-022-01108-1.

## Background

Male sexual dysfunction (SD) is defined as difficulty during any stage of the sexual encounter that prevents or impairs the individual or couple from enjoying sexual activity. This includes erectile, ejaculatory, and orgasmic dysfunction and hypoactive sexual desire disorder (HSDD) [1].

Erectile dysfunction (ED), being the commonest form of sexual dysfunction, is common among males with diabetes and is ranked as the third most important complication in diabetes [2]. The reported prevalence of SD in diabetes varies widely between studies from different parts of the world and depending on the study population [3]. The individual's response to SD would also differ remarkably depending on the social context.

Complex pathogenic factors are operating in diabetes to cause SD. The main recognised factors are vasculopathy, neuropathy, insulin resistance, visceral adiposity, hypogonadism, and endothelial dysfunction due to hyperglycaemia [4]. Contributing factors vary depending on the patient population studied, including the stage of the disease and socio-demographic factors.

Though many studies report the prevalence of different components of SD in men with diabetes, there is a dearth of data in a holistic context. There had been no in-depth assessment of associations, including mental health factors, hypogonadism, autonomic neuropathy, and vascular insufficiency. Assessment of its impact on patients' lives and their health-seeking pattern is further limited. Only a few studies assess the prevalence and associations of sexual dysfunction in Sri Lankan men with diabetes [5–7]. Therefore, it was considered timely to perform a comprehensive study on SD among men with diabetes to assess the prevalence of different components of SD (ED, orgasmic dysfunction, lack of desire), to identify its impact, the level of evaluation and treatment received by these men and to recognise associated factors of ED.

## Methodology

A Hospital-based Cross-sectional study was conducted from January to September 2020 at the Diabetes Clinic, National Hospital of Sri Lanka. Males with diabetes above the age of 18 years with the opportunity for sexual activity were included. Patients who have had genitourinary surgery or trauma that can affect the sexual function due to local anatomical factors and patients with major organ failure limiting sexual activity (Stage 5 CKD, decompensated liver disease, symptomatic congestive cardiac failure, prior stroke with residual limb weakness, dementia and psychotic state) were excluded.

Consecutive sampling was performed when the potential participants were obtaining appointments for the annual end-organ assessment. Socio-demographic information was collected using an interviewer-administered questionnaire. All three domains of SD were assessed as described below.

Erectile dysfunction was assessed using the short version of the International Index of Erectile Function (IIEF-5) [8]. Erectile dysfunction was diagnosed with a score below 22 out of 25, and graded as mild (17–21), mild to moderate (12–16), moderate (8–11), and severe (1–7). Sinhala and Tamil translations of this questionnaire used in a previous study in Sri Lanka were used with the authors' permission [6].

Premature ejaculation (PE) was assessed using the Premature Ejaculation Diagnostic Tool (PEDT) [9]. A score of 8 or less was considered normal, whereas 9–10 was considered probable PE and 11 or more as the presence of PE. This questionnaire was translated to Sinhala and Tamil by investigators having clinical experience and a professional translator and back-translated to assess the accuracy. After discussing the discrepancies, the questionnaires were revised and pre-tested using five clinic patients. After assessing participant response during pre-testing, the translations were finalised.

Libido was tested on a Likert scale by the interviewer: Very good, Good, Fair, Reduced and Very much reduced. The last two responses were taken as evidence of low libido.

The rest of the data collection was performed on the subsequent visit. Details of treatment-seeking behaviour for SD and health care providers' response were assessed by direct interviewing and referring to medical records. The effect of SD on their relationships and psychological status was assessed by direct questioning.

Details on diabetes, glycaemic control, co-morbidities, current medicines, and complications were collected using the participants' clinic records, including the investigations performed for the latest annual end-organ assessment. Routine end-organ assessment at the clinic includes clinically assessing for ischaemic heart disease, stroke and transient ischaemic attacks, and lower limb peripheral arterial disease (PAD). In addition, lower limb pulses and ankle-brachial pressure index (ABPI) were tested routinely. Absence of dorsalis pedis and posterior tibial pulse in at least one limb or ABPI < 0.81 was considered as examination findings of PAD [10].

Patients underwent dilated retinal photography and visual acuity assessment. Serum creatinine, estimated glomerular filtration rate (eGFR) and testing for proteinuria were performed to detect nephropathy. Peripheral neuropathy was assessed using a 10 g monofilament and vibration perception threshold assessment by biothesiometer. In addition, fasting, morning total testosterone was tested using the chemiluminescence method.

Short-form Health Survey-36 (SF-36) [11] was used to assess the quality of life (QoL). A previously validated Sri Lankan version was used [12]. Scoring of QoL was performed according to the standard guide under each component [13]. Mental health was assessed using the Depression, Anxiety and Stress Scale- 21 (DASS-21) [14]. This questionnaire was validated for Sri Lanka [15]. This was subsequently used in several Sri Lankan studies in Sinhala and Tamil in parallel with the psychiatrists' assessment improving the validity of the questionnaire [16].

Cardiovascular autonomic reflex tests (CART) using heart rate response to deep breathing, standing from supine position and Valsalva manoeuvre and blood pressure response to standing was performed [17–19]. Standard criteria were used to define cardiovascular autonomic neuropathy (CAN).

Colour Doppler ultrasonography (CDU) of the penis and cavernosal arteries was offered to participants with ED and performed if consent was given separately. Ultrasound of the flaccid penis was performed using a high frequency (7.5 Hz) linear probe. After assessing the paired corpora cavernosa, cavernosal arteries, tunica albuginea and corpus spongiosum, baseline peak systolic velocity (PSV) and end-diastolic velocity (EDV) of the cavernosal arteries were also obtained [20].

Pharmacological induction of erection was done using an intracavernosal injection of 60 mg papaverine near the penile base with a syringe under ultrasound guidance [21]. Post-injection measurements of the PSV and the EDV of the cavernosal arteries were taken at 5, 10, 15, and 20 minutes. Best PSV values < 25 cm/sec after the pharmacological induction was taken as evidence of arterial dysfunction whereas a value between 25–35 cm/s was considered borderline and > 35 cm/s was normal. End diastolic velocity > 5 cm/s during all phases of the erection indicates venous dysfunction in a patient with normal PSV [22].

### Sample size

Estimated sample size (N) was based on the following formula, *N* = 4 (Z)^2^ p (1-p) / D^2^ [23]. Standard normal deviate (Z _crit_) was taken as 1.96 for a CI of 95%. Based on the available literature, the pre-study estimate (p) for the prevalence of sexual dysfunction was taken as 0.73, and the total width of confidence interval (D) was 0.01092 [24]. This gave a sample size of 254 for the primary objective. To allow for 10% dropout, it was planned to recruit 280 participants. However, due to the COVID-19 outbreak, patient encounters were restricted at the clinic and the study was prematurely concluded after recruiting 212 participants.

### Data analysis

All data were entered into the Statistical Package of Social Sciences-19. Descriptive data were calculated using proportions and mean with standard deviation. Associations were assessed using the chi-square test or the Fisher’s exact test for proportions and the independent sample t-test for continuous variables.

## Results

Two hundred and twelve participants were enrolled in the study. Details of sexual dysfunction were available for all the participants. There were missing data on other sections due to restrictions to follow up the participants during the COVID-19 outbreak.

The median age was 56 years and 69 (32.5%) men were over 60 years. Characteristics of the study participants are described in Table 1.

**Table 1:** Characteristics of the study participants

Characteristics	Number of participants with available data	Distribution of the characteristics
Age (years)	212	Mean: 54.1 (SD: 10.1)
Civil status	212	Married: 204 Single: 8
Highest level of education	202	Less than grade 5: 6 Grade 5 to ordinary level: 44 Completed ordinary level: 95 Completed advanced level: 44 Received University education: 13
Ethnicity	209	Sinhala: 153 Tamil: 37 Muslim: 18 Other: 1
Smoking	177	Currently smoking: 27 Quit: 62 Never smoked: 88
Alcohol use	177	Currently using: 110 Quit: 34 Teetotaller: 33
Duration of diabetes (years)	179	9.9 (7.4)
Latest Fasting plasma glucose (mg/dL)	153	143.6 (53.3)
Latest HbA1C (%)	108	8.4 (1.8)
Body mass index (kg/m^2^)	125	25.8 (4.8)
Hypertension	177	Yes: 90 No: 87
Dyslipidaemia	176	Yes: 110 No: 66
Non-alcoholic fatty liver disease	154	Yes: 24 No: 130
Ischaemic heart disease	173	Yes: 17 No: 156
Lower limb peripheral arterial disease	144	Yes: 14 No: 129
Stroke/ Transient ischaemic attack	172	Yes: 0 No: 172
Chronic kidney disease	132	Yes: 42 No: 90
Diabetic retinopathy	140	Yes: 45 No: 95
Peripheral Neuropathy	140	Yes: 48 No: 92

**Fig. 1:**
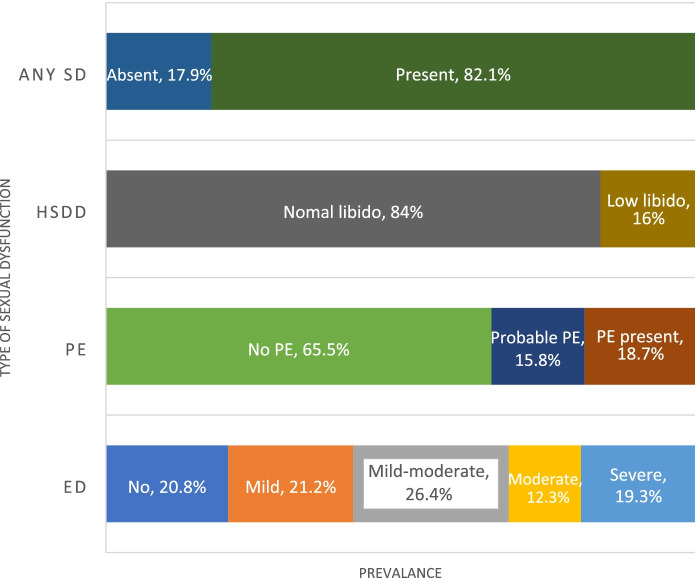
The prevalence and severity of sexual dysfunction among study participants

The prevalence and severity of sexual dysfunction among study participants are summarised in Fig. 1. Erectile dysfunction was more prevalent among men over 60 years (89.9%) compared to men aged 60 years or less (74.1%), (*p* = 0.008). However, the prevalence of low libido was not significantly different between older men (20.3%) and younger participants (14%), (*p* = 0.241). Premature ejaculation was not applicable in 73 since they could not achieve successful ejaculation or did not attempt sexual activity due to dysfunction in another domain. Percentages were calculated excluding them.

Duration of sexual dysfunction varied from 3 months to 22 years among the participants, with a mean of 4.6 (± 4.12) years.

Out of the 174 participants with SD, data on the effect of SD was available for 137 participants. There were both psychological and relationship effects on 36 (26.3%), whereas 45 (32.8%) had only psychological and 2 (1.5%) had only relationship effects. Thirty nine (62.9%) of the men over 60 years also experienced psychological and/or relationship issues due to SD which was not significantly different from their younger counterparts (*p* = 0.198).

Out of 174 participants with SD, 149 (85.6%) have never revealed SD to a health care provider. Out of the 25 participants who revealed, eight have revealed to a doctor in the diabetes clinic, 10 to their family physician and seven to another specialist. Only four participants were inquired by the health care provider about any sexual health issues, whereas all the others volunteered themselves. Fifteen have received treatment from a doctor, and three have taken medicines themselves. Out of those, only four participants experienced good responses. Five experienced a partial response, whereas nine others had no response to therapy.

Factors assessed for association with erectile dysfunction are summarised in Table 2 (continuous variables) and Table 3 (dichotomous variables).

**Table 2:** Associations of erectile dysfunction among the study participants (continuous variables)

Variable	Group with erectile dysfunction (*n* = 168)		Group without erectile dysfunction (*n* = 44)		Significance
	Number of respondents	Mean (SD)	Number of respondents	Mean (SD)	
Age (Years)	168	55.5 (9.5)	44	48.7 (10.6)	** < 0.001**
Duration of diabetes (Years)	144	10.9 (7.6)	35	5.89(4.7)	** < 0.001**
Latest Fasting plasma glucose (mg/dL)	121	139.1 (47.5)	32	160.7 (69.3)	0.104
Latest HbA1C (%)	86	8.3 (1.7)	22	8.8 (2.0)	0.212
Body mass index (kg/m^2^)	101	26 (4.9)	24	25.3 (4.1)	0.523
eGFR (ml/min/1.73m^2^)	73	73.9 (27.7)	9	100.5 (28.1)	**0.008**
Quality of life domains (SF-36)					
Physical functioning	43	64.2 (24.2)	8	75 (26.5)	0.258
Role-physical	43	63.9 (44.4)	8	84.4 (18.6)	**0.040**
Role- emotional	43	65.1 (43.1)	8	79.2 (30.5)	0.389
Vitality	43	51.1 (22.4)	8	63.1 (30.5)	0.332
Emotional well-being	43	58.6 (24.4)	8	62 (27.8)	0.723
Social functioning	43	76.7 (23.6)	8	71.9 (39.4)	0.634
Bodily pain	43	70.1 (28.6)	8	79.7 (30.6)	0.391
General health	43	49.2 (23.7)	8	55.6 (15.7)	0.459
Health change	43	44.2 (26)	8	53.1 (28.1)	0.382

**Table 3:** Associations of erectile dysfunction among the study participants (dichotomous variables)

Variables	Group with erectile dysfunction (*n* = 168)		Group without erectile dysfunction (*n* = 44)		Significance
	Number of respondents	Number (Percentage)	Number of respondents	Number (Percentage)	
Hypertension	142	73 (51.4)	35	17 (48.6)	0.764
Dyslipidaemia	139	88 (63.3)	36	21 (58.3)	0.583
Ischaemic heart disease^a^	139	15 (10.8)	34	2 (5.9)	0.309
Lower limb peripheral arterial disease^a^	115	14 (12.2)	28	0 (0)	**0.04**
Chronic kidney disease	107	37 (34.6)	25	5 (20)	0.159
Diabetic retinopathy	111	44 (39.6)	29	1 (3.4)	** < 0.001**
Peripheral neuropathy	112	43 (38.4)	28	5 (17.9)	**0.041**
Non-alcoholic fatty liver disease^a^	123	18 (14.6)	31	6 (19.4)	0.580
Current or past alcohol use	141	110 (78)	36	34 (94.4)	**0.024**
Current or past smoking	141	65 (46.1)	36	23 (63.9)	0.057
Low testosterone^a^	81	6 (7.4)	19	0 (0)	0.592
Abnormal cardiovascular autonomic function tests	105	75 (71.4)	23	15 (65.2)	0.555
Use of tricyclic anti-depressants^a^	130	14 (10.8)	30	0 (0)	**0.047**
Mental health parameters (DASS-21)					
Depression^a^	42	20 (47.6)	8	2 (25)	0.216
Anxiety^a^	42	22 (47.6)	8	2 (25)	0.151
Stress^a^	42	12 (28.6)	8	2 (25)	1

^a^Significance calculated using Fisher’s exact value

**Fig. 2:**
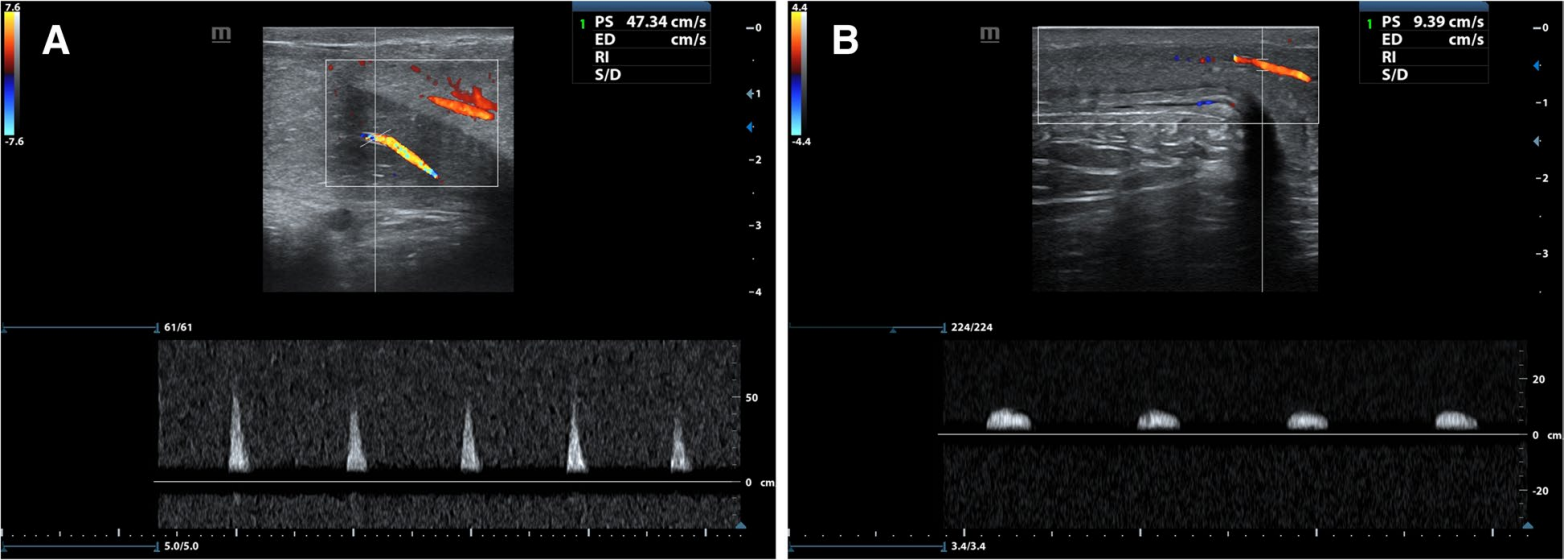
Longitudinal images acquired during penile Colour Doppler ultrasonography in two participants. Normal waveform with peak systolic velocity > 35 cm/s during the tumescent phase (**A**) in a participant without arterial insufficiency compared to a participant with peak systolic velocity < 25 cm/s (**B**)

Forty participants with ED underwent penile CDU to assess vascular aetiology for ED. Twenty participants had definite evidence of arterial insufficiency (PSV < 25 cm/s), whereas there was borderline flow (PSV 25–35 cm/s) in two participants and normal flow (PSV > 35 cm/s) in 18 participants. Images of CDU of two participants with a normal and an abnormal cavernosal arterial flow are shown in Fig. 2. A short video clip of the penile CDU of a participant showing good flow across cavernosal and helicine arteries is included as additional file 1. Hypertension was present in 75% of the participants with PSV < 25 cm/s whereas, only in 35% with PSV > 25 cm/s (*p* = 0.011). Clinical evidence of ischaemic heart disease or lower limb PAD could not predict abnormal flow in penile CDU. Only three out of 20 with low PSV had been diagnosed with ischaemic heart disease.

## Discussion

The reported prevalence of SD among men with diabetes varies significantly between different studies depending on the study setting and the method of diagnosing SD. In a meta-analysis of data from 145 studies on 88,577 men, the worldwide prevalence of ED in diabetes was estimated to be 52.5% (95% CI: 48.8–56.2) [3]. In the same meta-analysis, data from the Asian continent have revealed a prevalence of 67% (95% CI: 60–73.1).

One population-based study from Colombo revealed that 18.4% were experiencing SD of any type while 16.8% were experiencing ED [5]. Ejaculatory dysfunction and reduced libido were much less common. In another hospital-based study, ED was seen in more than 73.1% of the patients, while PE was noticed in 40.2% and low libido in 25% [24]. Another recent study has revealed that ED was seen in 63% of men with diabetes. This study did not report on the other aspects of sexual dysfunction [6].

Results of our study give the highest reported prevalence of ED in Sri Lanka. However, results seem to be comparable in the pattern of distribution in different modalities of SD. The higher prevalence in our study might be partly attributed to the study setting being a tertiary referral centre. As a result, our study participants are likely to represent a group with established complications and difficulty in achieving therapeutic goals even after excluding men with advanced complications limiting sexual activity.

The inclusion of older adults (> 60 years) could be considered a potential contributor to the high prevalence of SD in our study population. However, it is noteworthy that ageing alone contributes minimally to male SD in the absence of comorbidities [25]. Additionally, many similar studies done previously have recruited men up to 70 [26] or even 80 years [7, 27]. The pooled prevalence of ED among men with diabetes over 60 years of age according to a recent systematic review is 66.7 (95% CI: 57.5–74.8%) [3]. This is much lower than the figure obtained in our study. Rates for healthy older men vary widely with values between 11–86.3% according to the available literature. Most older men have considered it important to stay sexually active indicating the need to address their sexual health issues [25]. In our study also, older men have experienced psychological/ relationship issues due to SD similar to young men.

An alarming finding in our study was the lack of expression of SD to a health care provider despite over 60% having psychological and or relationship effects of SD. Nisahan et al. reported that 98.8% of their study participants had not been screened for SD [6]. Despite the psychological and relationship effect of SD, their reluctance to express SD could be due to stigma, busy clinic environment, and associated low self-esteem. This highlights the importance of screening at least selected men with diabetes for SD routinely. This would have provided them with an opportunity to express their sexual health issues. Particularly, in Asian culture, people are reluctant to voluntarily reveal their sexual health matters.

Our study showed that increased age and duration of diabetes, diabetic retinopathy, peripheral neuropathy, PAD, lower eGFR, and use of tricyclic anti-depressants (TCAD) are associated with ED. We did not assess associations with other domains of SD since their prevalence was lower than that of ED. Our study did not show any association with hypertension, CAN, clinical cardiovascular disease (CVD), or low testosterone. Any association with mental health parameters were also not evident. However, our study was not powered to identify those associations since the sample size was calculated for the first objective. Therefore, we cannot convincingly refute associations with those factors.

There is inconsistency in reported associations in the available literature. For example, one hospital-based study from Sri Lanka has shown an association with age > 40 years, duration of diabetes > 5 years, co-existing hypertension, and unsafe alcohol use to be associated with ED [6]. Another hospital-based study found lower income, duration of diabetes, older age, presence of hypertension and no alcohol intake to be associated with ED [7]. In contrast, another community-based study has shown that only the duration of diabetes is associated with SD [5]. According to a recent meta-analysis, continent, age, type of diabetes and method used to diagnose ED show significant associations [3]. Overall, our study also has replicated some common associations such as age, duration of diabetes and presence of other complications. This would enable the clinicians to target high-risk individuals to screen during clinical practice.

From a pathophysiological perspective and studies specifically assessing the association between vascular disease and ED in diabetes, we expect a strong correlation between vascular disease and ED. Further, ED is considered to represent an early manifestation of subclinical CVD [28]. Therefore, it is likely that cross-sectional studies can underestimate the association of clinical CVD with ED. Penile CDU is considered a valuable and minimally invasive tool in establishing vascular causes for ED [20]. It has been shown that diabetes-related ED is associated with poor response to cavernosal injection and poor flow, which is an indicator of vascular aetiology [29]. As shown from the small group of patients who underwent CDU in our study, arterial insufficiency is common in these patients with ED. The majority had no previously diagnosed CVD. Therefore, studies should be designed to see whether arterial insufficiency in CDU rather than ED itself could be used to predict future CVD. This would provide an opportunity for targeted primary prevention strategies for CVD.

Autonomic neuropathy is one contributing factor of SD and urogenital symptoms. Standardised tests to objectively diagnose pelvic autonomic neuropathy are not readily available. Therefore CART has been used as a surrogate marker of pelvic autonomic neuropathy [17]. There are mixed results though the trend is toward SD being associated with CAN [30, 31]. We did not observe this association in our study. This could be due to lack of power, higher prevalence of CAN observed in our patients and multifactorial pathophysiology of ED in diabetes. One striking observation was the very high rates of CAN in our study population compared to the other studies [32]. Since assessing CAN was not a primary objective of our study, we could not draw any conclusions on this. Further studies are warranted to verify and investigate the clinical implications of this observation.

Hypogonadism is recognised to be associated with diabetes through various postulated mechanisms. Some studies have shown that SD in men with diabetes is associated with hypogonadism [33, 34]. We could not show any significant association due to the small number of patients with low testosterone. But all the patients with low testosterone in our study had ED. This emphasises the current recommendation to test total testosterone level in ED associated with diabetes.

Studies have shown that SD causes a significant impact on QoL in patients, including studies from Sri Lanka [24]. There had been concerns of loss of masculinity, reduced intimacy and partner mistrust leading to complex psychosocial and relationship issues [35]. In our study, we found an association with the role-physical domain of the SF-36 tool. We cannot determine the cause-effect relationship since this could also be due to associated other complications in patients' ED.

Limitations of our study include the inability to complete the intended sample size and missing data on several variables. This was inevitable due to the impact of the COVID-19 pandemic, which hindered the routine outpatient visits and annual end-organ screening at the diabetes clinic. Therefore, we had to conclude the study prematurely. However, our study design has allowed comprehensive evaluation of at least a portion of the patients assessing many aspects related to SD. Future studies should perform a comprehensive evaluation of a larger sample size to understand the interrelationship between these factors better. In addition, follow-up of these patients to detect the development of macrovascular complications is warranted so that associations can be recognised with penile CDU findings.

## Conclusions

Our study has shown an alarmingly high prevalence of SD among men with diabetes, which has been under-recognised and grossly undertreated despite its psychological and relationship impact. Erectile dysfunction is associated with increased age, duration of diabetes and accumulating complications. An in-depth assessment of aetiology and effects is unrealistic from a cross-sectional study due to the complex interplay between multiple factors. Follow-up studies are required to define these complex interrelationships.

## Supplementary Information

**Additional file 1.** A video clip of a longitudinal view of the penile colour Doppler ultrasonography of the ventral aspect of the cavernosal bodies in a participant with good arterial flow. Normal flow through cavernosal and helicinearteries is visualised.

## Data Availability

The datasets used during the current study are available from the corresponding author on reasonable request.

## Abbreviations

ABPI: Ankle-brachial pressure index
CAN: Cardiovascular autonomic neuropathy
CART: Cardiovascular autonomic reflex tests
CVD: Cardiovascular disease
CKD: Chronic kidney disease
CDU: Colour Doppler ultrasonography
EDV: End-diastolic velocity
ED: Erectile dysfunction
eGFR: Estimated glomerular filtration rate
HSDD: Hypoactive sexual desire disorder
IIEF-5: Short version of the International Index of Erectile Function
PAD: Peripheral arterial disease
PSV: Peak systolic velocity
PE: Premature ejaculation
PEDT: Premature Ejaculation Diagnostic Tool
QoL: Quality of life
SD: Sexual dysfunction
